# Effective and realistic sequestration of Sr^2+^ and B^3+^ ions from the aqueous environments using coral reefs based Ca-MCM-41: Gulf of Suez as case study

**DOI:** 10.3389/fchem.2025.1550726

**Published:** 2025-04-16

**Authors:** Alshaima Sayed, Ahmed M. El-Sherbeeny, Gouda Ismail Abdel-Gawad, Essam A. Mohamed, Wail Al Zoubi, Mostafa R. Abukhadra

**Affiliations:** ^1^ Faculty of Earth Science, Beni-Suef University, Beni-Suef, Egypt; ^2^ Materials Technologies and their applications Lab, Geology Department, Faculty of Science, Beni-Suef University, Beni Suef, Egypt; ^3^ Industrial Engineering Department, College of Engineering, King Saud University, Riyadh, Saudi Arabia; ^4^ Applied Science Research Center, Applied Science Private University, Amman, Jordan; ^**5**^ Materials Electrochemistry Laboratory, School of Materials Science and Engineering, Yeungnam University, Gyeongsan, Republic of Korea

**Keywords:** coral reefs, Ca-MCM-41, strontium, boron, adsorption, advanced modeling, seawater

## Abstract

A mesoporous calcium-bearing siliceous framework (Ca-MCM-41) was synthesized using natural coral reef carbonate rocks as precursors. The structural characterization, confirmed through XRD, SEM, FT-IR, and BET analyses, validated the formation of the MCM-41 framework with well-defined mesoporous properties and a high surface area of 159.6 m^2^/g. The developed Ca-MCM-41 was evaluated as a potential adsorbent for the removal of Sr^2+^ and B^3+^ ions from both aqueous solutions and real seawater samples collected from the Gulf of Suez, Egypt. The adsorption capacity at saturation reached 285.9 mg/g for Sr^2+^ and 86.1 mg/g for B^3+^, demonstrating the framework’s high affinity for these contaminants. The adsorption mechanisms were elucidated using steric and energetic parameters, as derived from statistical physics-based isotherm models. The Ca-MCM-41 framework exhibited a higher active site density (148.9 mg/g) for Sr^2+^ compared to B^3+^ (54.8 mg/g), explaining its superior sequestration performance for strontium ions. Each receptor site was capable of accommodating up to three Sr^2+^ ions and 2 B^3+^ ions, indicating a multi-ionic interaction process and preferential vertical alignment during adsorption. Energetic analysis revealed that the sequestration process occurred via physical adsorption with interaction energies below 7 kJ/mol, alongside exothermic and spontaneous behavior, as evidenced by calculated internal energy, entropy, and enthalpy values. The developed Ca-MCM-41 structure demonstrated notable efficiency in real seawater applications, achieving sequestration percentages of 80% for Sr^2+^ and 64% for B^3+^, considering their average concentrations (24.2 mg/L for Sr^2+^ and 12.85 mg/L for B^3+^) in a 1-L volume. These findings highlight the high potential of Ca-MCM-41 as an effective and sustainable adsorbent for Sr^2+^ and B^3+^ removal in environmental water treatment applications.

## 1 Introduction

The contamination of water resources with hazardous chemicals represents a severe environmental and public health challenge, threatening long-term ecological stability and human wellbeing (Dehkordi et al., 2024). Among the primary sources of water pollution are unregulated discharges from industrial, agricultural, and mining activities, which introduce highly toxic and persistent contaminants into aquatic ecosystems (Zourou et al., 2022; Arab et al., 2022). The presence of hazardous metal ions and other pollutants in water, either in free ionic form or complexed with other compounds, poses significant risks to biodiversity and human health (Zourou et al., 2022; Ighnih et al., 2024). Many of these ions are highly toxic, non-biodegradable, and bioaccumulative, with some exhibiting carcinogenic properties and a tendency to accumulate in human and animal tissues over time (Ighnih et al., 2024; Kusuma et al., 2024; Wu et al., 2024). Key contributors to this form of contamination include metallurgical industries, chemical extraction processes, and nuclear fuel production, all of which release substantial amounts of cadmium, boron, arsenic, zinc, barium, cobalt, chromium, lead, and strontium into water systems (Bayar et al., 2024; Shi et al., 2019; Che et al., 2024). Among these, the increasing prevalence of boron (B^3+^) and strontium (Sr^2+^) pollution in desalination processes has become a topic of heightened concern in recent years.

Boron, a non-metallic element, is widely utilized in glass manufacturing, semiconductors, fertilizers, pharmaceuticals, and nuclear energy applications (Dong et al., 2023). Its prevalence in water bodies is primarily attributed to the extensive industrial use of boron-containing materials, leading to elevated concentrations in aquatic environments (Afolabi et al., 2021; Liu T. et al., 2020). Although boron is an essential micronutrient for plants and animals, excessive exposure can disrupt biological functions and impact ecosystem balance (Duran et al., 2022). Prolonged exposure to boron-contaminated water has been linked to neurological and reproductive disorders, along with adverse effects on the digestive and endocrine systems in humans (Bai et al., 2023; Hong et al., 2021; Rahman et al., 2021). Symptoms of boron toxicity include gastrointestinal distress, hormonal imbalances, and developmental complications such as pregnancy-related disorders and congenital anomalies (Nielsen, 2014). The World Health Organization (WHO) has set maximum permissible limits for boron in drinking water at 2.4 ppm and 1 ppm for irrigation water to prevent toxicity in agricultural crops, as excessive boron levels can impair photosynthetic efficiency and crop yield (Dong et al., 2023; Hassan et al., 2024; Wang et al., 2021; Kim and Kim, 2023). A major challenge in boron removal arises in seawater desalination, where boron exists primarily in the uncharged boric acid (H_3_BO_3_) form, which exhibits high chemical stability and low affinity for conventional removal techniques (Dong et al., 2023; Bai et al., 2023; Wang et al., 2021). Reverse osmosis (RO), the most widely used desalination technology, is often inefficient in completely removing boron due to its small atomic size and low ionization under typical desalination conditions. Additionally, boron concentrations in the treated water often fluctuate beyond safe drinking levels, depending on operational factors and membrane efficiency (Ruiz-García et al., 2018). As a result, alternative and more effective strategies for boron removal are critically needed (Dong et al., 2023).

Strontium ions (Sr^2+^) are naturally present in groundwater and seawater, typically at concentrations of 6–7 mg/L (Nur et al., 2017; Pepper et al., 2024). However, the environmental impact of strontium pollution gained significant attention following the Fukushima nuclear disaster, which led to the release of radioactive strontium-90 (^90^Sr) into marine ecosystems (Pepper et al., 2024; Al-Absi et al., 2022). This radionuclide is considered one of the most hazardous contaminants due to its high solubility, bioavailability, and ability to integrate into biological systems (Nur et al., 2017; Al-Absi et al., 2022). Strontium has numerous industrial applications, including its use in glass production, ceramics, pyrotechnics, and fluorescent lighting (Nur et al., 2017; Choi et al., 2021). However, its radioactive isotopes pose severe health risks, particularly through their ability to mimic calcium and integrate into bone tissue, leading to skeletal abnormalities and increased risks of bone cancer and soft tissue malignancies (Al-Absi et al., 2022; Yang et al., 2020). In addition to its radiotoxic effects, non-radioactive Sr^2+^ at elevated levels in drinking water has been associated with neurological disorders, cognitive impairments, and developmental deficits (Nur et al., 2017; Eun et al., 2021; Huo et al., 2021). The WHO and the U.S. Environmental Protection Agency (EPA) have established regulatory limits for Sr^2+^ concentrations in drinking water between 1.5 mg/L and 4.2 mg/L to minimize health risks (Al-Absi et al., 2022). Furthermore, Sr^2+^ ions pose operational challenges in seawater desalination, as they interact with carbonate (CO_3_^2-^) and sulfate (SO_4_^2-^) ions, leading to scaling and membrane fouling in reverse osmosis systems, thereby reducing their efficiency (Reddy et al., 2021).

Various methods have been explored for removing boron and strontium from water, including adsorption, nanofiltration, ion exchange, coagulation, flocculation, and membrane-based separation techniques (Feng et al., 2024; Wang et al., 2024; Li Y. et al., 2024). Among these, adsorption-based approaches have gained significant attention due to their cost-effectiveness, simplicity, and environmental sustainability (Zhang P. et al., 2024; Noormohammadi et al., 2024; Karakoç and Erçağ, 2024). Recent research has demonstrated that nanostructured materials offer exceptional adsorption capabilities, making them highly suitable for removing metal ions from water (Naat et al., 2021; Al-Amrani and Onaizi, 2024). Several parameters influence the effectiveness of these adsorbents, including surface area, binding efficiency, regeneration potential, adsorption kinetics, and selectivity (Fan et al., 2024; Faheem et al., 2024). Consequently, there has been a strong emphasis on developing novel, low-cost, and naturally derived adsorbents to enhance water purification technologies (Ifa et al., 2022; Sun et al., 2024). Natural materials such as rock-based minerals and silica mesoporous nanoparticles have gained prominence due to their economic and ecological advantages (Chen et al., 2021). Among these, mesoporous silica materials, particularly MCM-41, have emerged as promising adsorbents due to their highly ordered porous structure (2–50 nm), large surface area, and excellent thermal stability (Li C. et al., 2024; Zhang S. et al., 2024). Additionally, the presence of silanol (-SiOH) functional groups within the silica framework enhances adsorption interactions with metal ions, significantly improving their removal efficiency (Guo et al., 2024; Sayed et al., 2025; Khan and Shaily, 2022; AbuKhadra et al., 2020).

The current research presents a novel approach for the efficient adsorption of Sr^2+^ and B^3+^ ions using calcium-modified mesoporous MCM-41, synthesized from naturally occurring carbonate-rich coral reef rocks. Our previous investigation demonstrates significant impact for the calcium precursor on the resulted MCM-41 modified derivatives (Sayed et al., 2025). The study integrates experimental characterization with advanced theoretical modeling, based on statistical physics principles, to optimize the adsorption mechanism. Key highlights of this study include (A) development of a calcium-functionalized mesoporous MCM-41 adsorbent, leveraging naturally available precursors to enhance environmental sustainability, (B) comprehensive equilibrium analysis, incorporating steric (saturating adsorption capacity, site occupation) and energetic (adsorption energy, internal energy, enthalpy, and entropy) parameters to gain deeper insights into adsorption behavior, and (C) application of the developed adsorbent for real seawater samples, collected from contaminated sites along the western Gulf of Suez, demonstrating its practical effectiveness in real-world conditions.

## 2 Experimental work

### 2.1 Materials

Natural coral reef-enriched carbonate rock specimens were extracted from an old rock unit in the Eastern Desert of Egypt. Cetyl-trimethylammonium bromide (CTAB) (98%), sulfuric acid (98%), ethanol (96%), and sodium silicate were bought via Sigma-Aldrich, Egypt, for the synthesis of mesoporous calcium silicates. Strontium (Sr(NO_3_)_2_ in 0.5 mol/L HNO_3_;1,000 mg/L Sr; NSPSC Code: 41116107) and boron (H_3_BO_3_ in H_2_O; 1,000 mg/L B; CAS Number:10,043-35-3) ICP standard solutions were delivered from the Sigma-Aldrich company and applied in preparing the polluted aqueous solution. Distilled water was used in the synthesis of the materials and the preparation of the contaminated aqueous solutions.

### 2.2 Synthesis of Ca-MCM-41

The production of MCM-41 nanostructures implementing coral reef as a calcium precursor was conducted according to the procedures provided by (Sayed et al., 2025). The coral reef carbonate samples were initially pulverized to attain a particle size range of 50–150 µm. The powdered product (100 g) was completely dissolved in 100 mL of H_2_SO_4_. This occurred for 24 h utilizing a magnetic stirrer at a maintained speed of 650 rpm. The resulting calcium sulfate solution was then extracted by a filtration process and removal of the residual solid phases and was then mixed with methanol (100 mL). After that, a solution of sodium silicate (20 g; 100 mL distilled water) was next poured into the reactor, which was followed by the incorporation of CTAB, while maintaining continuous stirring over 48 h at 1,000 rpm and 60 °C. The generated material was recovered out of the remaining solution by filtering and underwent thorough washing and neutralization before being dried at 70°C for 12 h. The sample underwent thermal treatment at 650 °C for 5 h to eliminate the residual surfactant. The product was designated as Ca-MCM-41.

### 2.3 Characterization instruments

The crystalline aspects and structural features were evaluated employing X-ray diffraction (XRD) patterns obtained from the PANalytical-Empyrean X-ray diffractometer. The diffractometer’s detection ranges within two theta angles extend between 0° and 70° for high-angle analysis and from 0° to 5° for low-angle analysis. The alteration of the key chemical groups during the manufacturing stages was assessed employing a Shimadzu FTIR-8400S spectrometer that possesses a measurement range of 400–4,000 cm^-1^. The surface characteristics of the fabricated materials were examined utilizing a Gemini Zeiss Ultra 55 scanning electron microscope. Prior to analysis, the exterior of the product was prepared by spraying it with a thin gold film as a coating layer. The porosity levels and specific surface area were measured using a surface area analyzer (Beckman Coulter SA3100) immediately after the removal of gases out of the sample. The analyses were conducted by employing the standard N_2_ adsorption and desorption isotherms.

### 2.4 Batch adsorption experiments

Adsorption experiments of Sr^2+^ and B^3+^ employing synthesized coral reef-based Ca-MCM-41 were performed in a batch mode, investigating the effects of pH (3–9), starting content (25–250 mg/L), and adsorption duration (30–720 min). The experiments occurred in three separate examinations, with an identical volume of 50 mL and a solid dose of 0.2 g/L. The adsorption equilibrium investigations were performed at various temperatures: 303 K, 313 K, and 323 K. Upon reaching the equilibrium stage of each test, the analyzed solutions were subjected to filtering to get rid of the solid particles of Ca-MCM-41, therefore detecting the remaining levels of Sr^2+^ and B^3+^. The remaining concentrations were analytically measured by implementing inductively coupled plasma mass spectrometry (Perkin Elmer). The Sr^2+^ (Sr(NO_3_)_2_ in HNO_3_ 0.5 mol/L 1000 mg/L Sr Certipur^®^; Catalogue Number: 119,799) and B^3+^ (H_3_BO_3_ in H_2_O 1000 mg/L B Certipur^®^; Catalogue Number: 119,500) standards utilized during the calibration procedures were obtained from Merck Company (Germany) and afterwards authorized by the National Institute of Standards and Technology (NIST). The results were subsequently applied to evaluate the adsorption characteristics of Ca-MCM-41, in line with Equation 1. The parameters Q_e_, C_o_, Ce, V, and m in the equation represent the adsorption capacity (in mg/g), the starting concentrations (in mg/L) of the analyzed ions, the residual concentrations (in mg/L) of the metal ions, the volume (in mL) of the contaminated solutions containing Sr^2+^ and B^3+^ ions, and the dose (in mg) of Ca-MCM-41.

$$Q_{e\;\left(mg/g\right)}=\frac{\left(C_{o}-C_{e}\right)V}{m}$$ (1)

### 2.5 Conventional and modern equilibrium investigations

The adsorption of Sr^2+^ and B^3+^ using synthetic coral reef-based Ca-MCM-41 has been discussed using established traditional kinetics, traditional equilibrium, and recent isotherm investigations in accordance with the mathematical concepts of statistical physics (Supplementary Table S1). The kinetic and classical equilibrium models were assessed employing non-linear fitting of the retention results for Sr^2+^ and B^3+^. The evaluation includes standard fitting variables, including the coefficient of determination (R^2^) (Equation 2) and Chi-squared (χ^2^) (Equation 3). The nonlinear matching properties of recent equilibrium model equations and the uptake results of Sr^2+^ and B^3+^ were evaluated based on the determination coefficient (R^2^) and root mean square error (RMSE) (Equation 4). The parameters m′, p, Qi_cal_, and Qi_exp_ in the equation represent the uptake outcomes, parameters influencing the retention, expected uptake capacity, and confirmed uptake capacities, respectively.

$$R^{2}=1-\frac{\sum {\left(Q_{e,\exp}-Q_{e,cal}\right)}^{2}}{\sum {\left(Q_{e,\exp}-Q_{e,mean}\right)}^{2}}$$ (2)

$$\chi^{2}=\sum \frac{{\left(Q_{e,\exp}-Q_{e,cal}\right)}^{2}}{Q_{e,\text{cal}}}$$ (3)

$$\text{RMSE}=\sqrt{\frac{\sum_{i=1}^{m}{\left({\text{Qi}}_{\text{cal}}-{\text{Qi}}_{\exp}\right)}^{2}}{m^{\prime }-p}}$$ (4)

### 2.6 Studied polluted area and sampling

**FIGURE 1:**
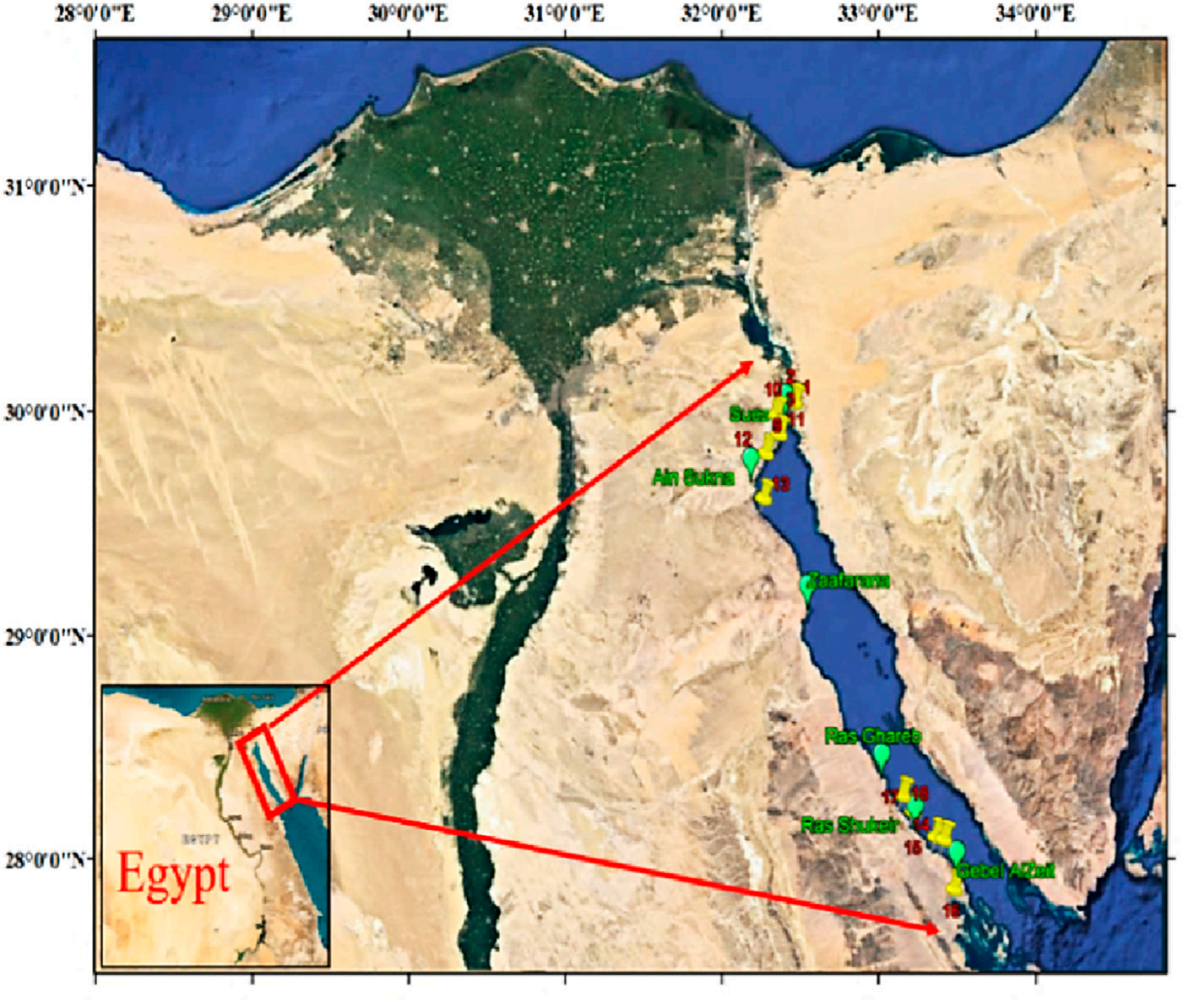
Location map for the area of study.

The Gulf of Suez (GOS) represents a semi-enclosed shallow basin extending 300 km between Port Suez and Shadwan Island, connected to the northern Red Sea via the Straits of Gubal and to the Mediterranean Sea through the Suez Canal. It is the biggest supplier of crude oil in Egypt, and consequently, numerous oil spills occurred in the Gulf, negatively impacting the coastal and marine environments. Furthermore, these regions are affected by elevated levels of various inorganic ions, including sulfate, strontium, boron, barium, and zinc, which impede successful desalination efforts and potential development strategies. Water samples were collected from eighteen locations throughout Egypt’s Gulf of Suez’s western shore (Figure 1). After being disinfected using diluted nitric acid and rinsed with sterile water, the samples were stored in polypropylene containers. This step was accomplished to prevent the deposition or possible adsorption of already present waterborne pollutants on the inner walls of the container. These samples were then stored in the refrigerator at around 4 °C to protect them from the effects of evaporation.

## 3 Results and discussion

This section presents a comprehensive evaluation of the synthesized Ca-MCM-41 material, encompassing its structural characterization, adsorption performance for Sr^2+^ and B^3+^ ions, kinetic and equilibrium modeling, and performance in realistic seawater matrices. Each subsection addresses key experimental outcomes in detail.

### 3.1 Characterization of the adsorbent

**FIGURE 2:**
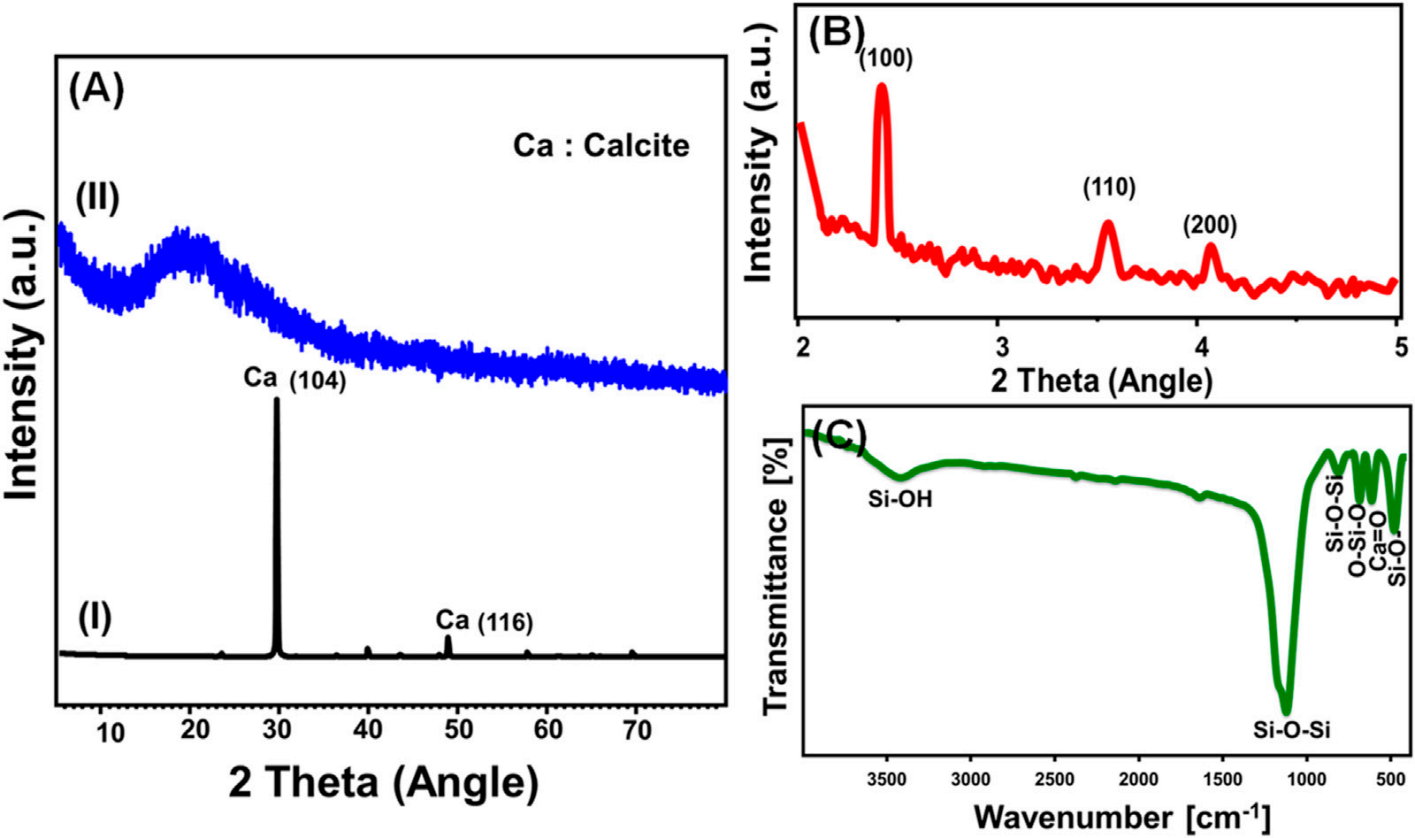
Shows **(A)** high angle XRD pattern of raw carbonate coral reefs (I) and high angle pattern of Ca-MCM-41 (II), **(B)** low angle pattern of Ca-MCM-41; and **(C)** FT-IR spectrum of Ca-MCM-41.

The structural characteristics were evaluated using high-angle and low-angle XRD patterns. The high-angle analysis of the implemented coral reef carbonate as a calcium precursor reveals its mineralogical nature as pure and highly crystalline calcite mineral with a CaCO_3_ chemical formula (ICDD; PDF No., 01-072-1973) (Figure 2A.I). The determined pattern of the produced Ca-MCM-41 framework employing coral reefs proves the effective integration of calcium into the mesoporous siliceous skeleton of MCM-41 (Figure 2A.II). The high-angle pattern of the synthesized Ca-MCM-41 exhibited only wide peaks at 22°, indicative of the characteristic peak of amorphous silica that comprises the Ca-MCM-41 framework (Sayed et al., 2025) (Figure 2A.II). The low angle pattern reveals that the obtained product exhibits the characteristic patterns of the MCM-41 form (Figure 2B). The recognizable peaks correlating with the crystallographic planes of (100), (110), and (200) of the Ca-MCM-41 structure were clearly detected in the obtained diffraction pattern (Figure 2B) (Sayed et al., 2025) (ICDD; PDF No., 00-049-1712).

The FT-IR spectrum was used to analyze the chemical composition of the synthesized Ca-MCM-41 derived from coral reefs, focusing on the vital structural groups that serve as principal active receptors throughout the adsorption reactions (Figure 2B). The pronounced absorption band at 1,100 cm^-1^ denotes the asymmetrical stretching of the Si-O-Si bonds, whereas the notable low-intensity bands at approximately 800 cm^-1^ and 680 cm^-1^ correspond to the symmetrical stretching of Si-O-Si and O-Si-O, respectively (Figure 2C) (Sayed et al., 2025; Matkala et al., 2023; Şimşek et al., 2023). The Si-O- group indicative of the MCM-41 mesoporous framework was detected by its matching band at 470 cm^-1^, whereas the principal reactive silanol group (Si-OH) was recognized through its distinct band at 3,400 cm^-1^ (Figure 2C) (Zhang et al., 2019). The presence of the Ca=O verification band (about 605 cm^-1^) validates the effective integration of calcium ions into the MCM-41 structure (Figure 2C) (Sayed et al., 2025; Choudhary et al., 2015). The chemical results were corroborated by the detected elemental compositions of Ca-MCM-41 according to its EDX spectrum (Supplementary Figure S1). The spectrum reveals its composition of Si (43.90%), Ca (15.65%), and O (40.45%).

**FIGURE 3:**
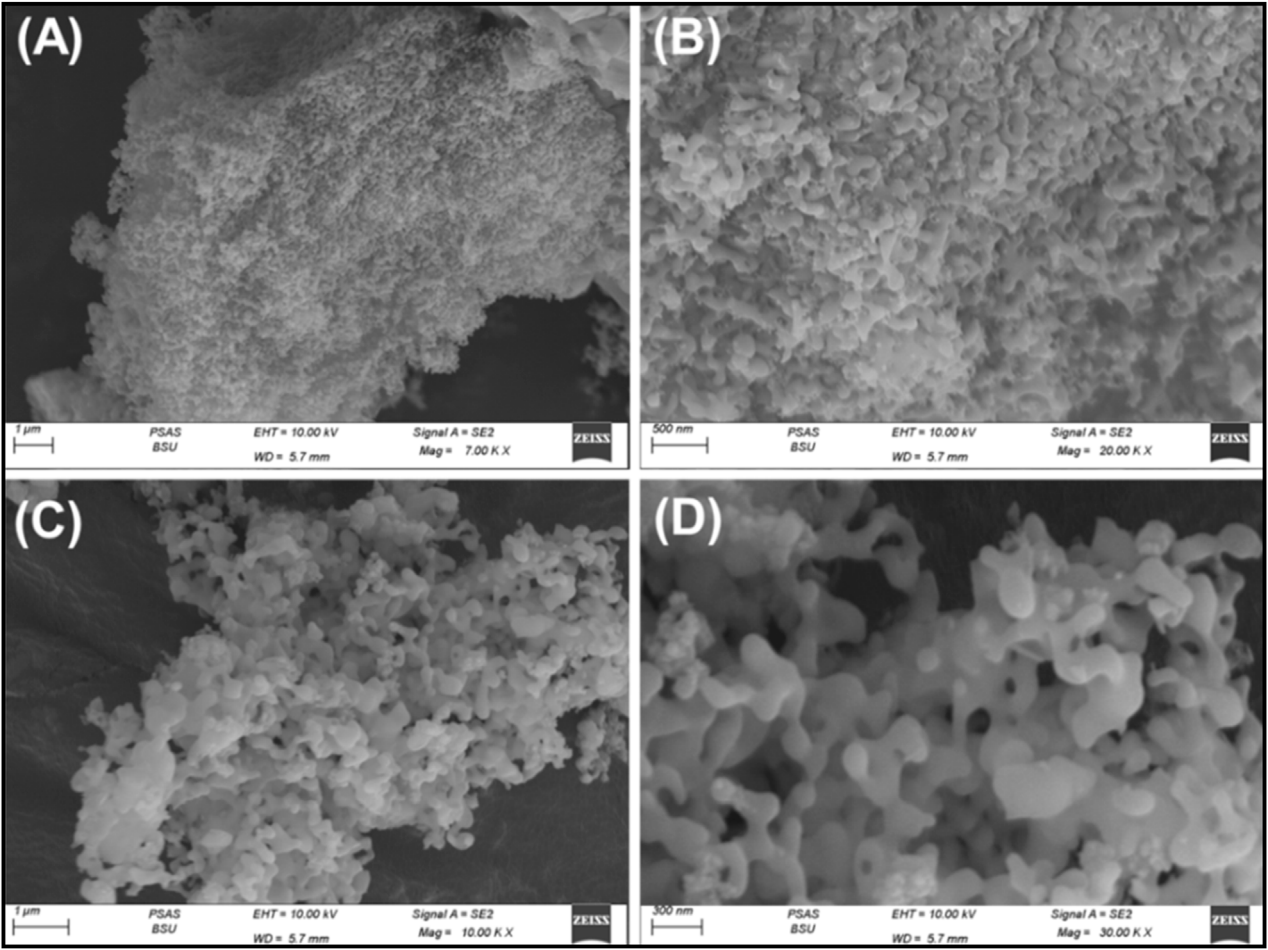
SEM images of the synthesized Ca-MCM-41 mesoporous structure using coral reefs carbonate **(A–D)**.

SEM images of the synthesized Ca-MCM-41 nanoparticles displayed their generation as a series of intersecting and interconnecting wavy or worm-like nano-grains, resulting in significantly rough and irregular surface features (Sayed et al., 2025) (Figures 3A, B). This surface mostly contains disconnected and connected interwoven pores, which serve as extra nanopores, hence augmenting the particles' porosity characteristics and interacting interface (Figures 3C, D). This was evident in the evaluated textural parameters, involving both porosity and surface area, based on the acquired N_2_ adsorption/desorption isotherm curve (Supplementary Figure S2). The isotherm curve is classified as type IV according to IUPAC classification, exhibiting a sharp ascent at high relative pressures (P/P_0_ > 0.8), indicating capillary condensation, which signifies the mesoporous characteristics of the material (Supplementary Figure S1) (Sayed et al., 2025; Liu Y. et al., 2020; Das et al., 2019). The curve exhibits a pronounced H1 hysteresis loop which is associated with well-defined cylindrical mesopores and characterizes the ordered mesoporous materials, such as MCM-41 or SBA-15, which have well-defined, interconnected cylindrical pores. The presence of a steep capillary condensation step suggests mesoporous materials (2–50 nm pore size). Therefore, the structure exhibits mesoporous properties including two-dimensional interior cylindrical tunnels with a surface area of 159.6 m^2^/g (Sayed et al., 2025; Das et al., 2019).

### 3.2 Adsorption results

#### 3.2.1 Effect of pH

**FIGURE 4:**
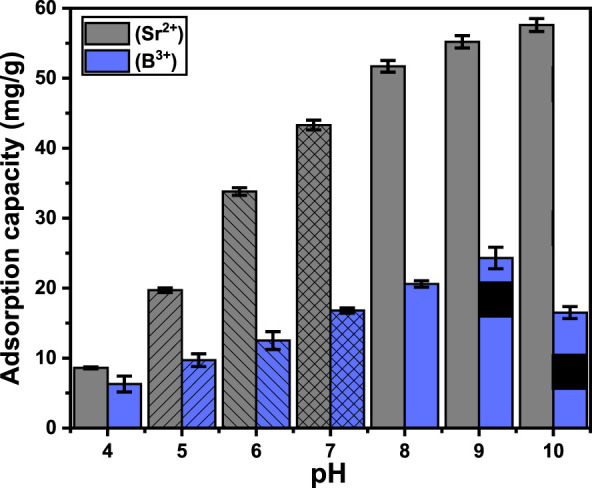
The experimental influence of pH on the uptake of Sr^2+^ and B^3+^ using Ca-MCM-41.

The pH of the aqueous solution significantly influences the essential distributed charges on the exteriors of the MCM-41 materials and the ionizing characteristics of water-soluble chemicals (Yaqub et al., 2024). The test’s influences of pH were established throughout pH ranges from 4 to 10. The other variables were kept constant: a time frame of 24 h, temperature of about 303 K, volume of 50 mL, metal concentration of 100 mg/L, and dosage of 0.3 g/L. The findings indicated a substantial increase in Sr^2+^ retention when the pH value rose from 4 (8.6 mg/g) to 10 (57.6 mg/g) (Figure 4). The ionizing or speciation characteristics of strontium at varying pH values, together with the impact of pH on the prevailing distribution of charges onto the exterior of Ca-MCM-41, may explain the observed phenomenon. The specified characteristics of Sr (II) indicate its presence as a positive ion (Sr^2+^) throughout aqueous environments with pH ranging from 4 to 10, with the ignored identification of SrOH^+^ and SrCl^+^ compounds (Suresh et al., 2024; Mironyuk et al., 2019). Ions with positive charges at elevated pH values are significantly influenced by the electrostatic attraction toward the exterior chemical groups of Ca-MCM-41, which have been modified by deprotonation processes, resulting in increased negative charges associated with the concentrated OH groups on their outermost surfaces. Therefore, when pH rises, the removal effectiveness improves in contrast to the acidic condition, where the protonation along with the accumulation of hydronium ions augments the electrostatic repulsion that already exists with strontium ions (Salam et al., 2020a).

The increase in pH from four to nine significantly enhances B^3+^ elimination from 6.3 mg/g to 24.3 mg/g, respectively. A further rise in examining pH had a reverse impact, leading to a considerable drop in the uptake effectiveness of B^3+^ using Ca-MCM-41 (Figure 4). Boron mostly exists in water as boric acid (H_3_BO_3_) together with borate (B(OH)_4_^-^) when its level is below 270 mg/L (Isaacs-Paez et al., 2014; Hu et al., 2020). Boric acid comprises a weak acid possessing a pKa of 9.2, functioning as a monoprotic Lewis acid that interacts with water, consuming a hydroxyl ion and emitting a proton, resulting in the borate ions (Isaacs-Paez et al., 2014; Amor et al., 2014). At a pH range spanning four to 6, boron mostly occurs as B(OH)_3_ (Hu et al., 2020). Consequently, there was minimal influence from the electrostatic attraction on the uptake of boron throughout this pH zone. As the pH rises from six to 9, B(OH)_3_ progressively converts into a B(OH)_4_^-^ form. At a pH of around 9.2, equivalent to the pKa value, the ratio of boron ions is 50% H_3_BO_3_ and 50% B(OH)_4_^-^. The optimal elimination of boron occurred around pH = 8, as a portion of boron existed as B(OH)_4_^-^, which was attracted to the remaining positive charges across the exterior of Ca-MCM-41. Although Ca-MCM-41’s surface charge is negative at pH > 8 up to 9.2, chelation involving the OH groups on its exterior and soluble orthoborate forms is still feasible (Amor et al., 2014), leading to a considerable binding. Above pH 9.2, H_3_BO_3_ is almost entirely converted into B(OH)_4_^-^ along with polyanionic forms (B_3_O_3_(OH)_4_^-^ and B_3_O_3_(OH)_5_^2-^) (Isaacs-Paez et al., 2014; Xu et al., 2012). These forms exhibit robust electrostatic repulsive forces with the strongly negatively charged exterior of Ca-MCM-41 and demonstrate considerable competition with the hydroxyl anions abundantly available in the solution.

#### 3.2.2 Contact time

**FIGURE 5:**
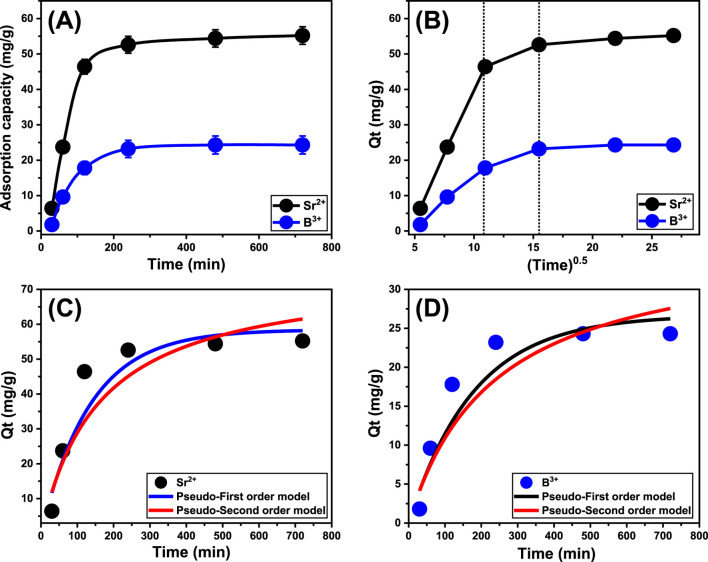
The effect of contact time on the uptake of Sr^2+^ and B^3+^ **(A)**, the Intra-particle diffusion curves of the uptake processes **(B)**, fitting of the Sr^2+^ uptake behaviors with the kinetic models **(C)**, and fitting of the B^3+^ uptake behaviors with the kinetic models **(D)**.

An experiment was conducted to assess the adsorption characteristics of Ca-MCM-41 regarding its efficacy in eliminating Sr^2+^ and B^3+^ ions. The assessment extends between 30 and 720 min. After establishing the levels of essential factors, including metal content (50 mg/L), temperature (303 K), volume (50 mL), pH (9), and quantity (0.2 g/L) at fixed values, the specific impacts of varied time periods were evaluated. The experiments revealed a significant increase in the effectiveness of Ca-MCM-41 during the uptake of Sr^2+^ and B^3+^, based on the amounts of sequestered ions and the associated actual retention rates (Figure 5A). Moreover, it is crucial to observe that the time span of the investigations significantly influences the measurable enhancements in the recognized uptake qualities, extending to about 240 min (Figure 5A). However, no notable changes or improvements were noticed in the elimination rate of Sr^2+^ and B^3+^ or the amount of these ions adsorbed after the prescribed contact periods. These evidences suggest that Ca-MCM-41 could potentially act as an adsorbent for Sr^2+^ and B^3+^, attaining maximal equilibrium after 480 min. During such equilibrium conditions, the adsorption capacities of Ca-MCM-41 were measured to be 55.2 mg/g for Sr^2+^ and 24.3 mg/g for B^3+^ (Figure 5A). Within the first steps of the testing, significant improvements in the binding and adsorption features of Sr^2+^ and B^3+^ were detected using the Ca-MCM-41 adsorbent, together with elevated amounts of immobilized ions. The highlighted increases were ascribed to the extensive distribution of both active and unfilled sites within the surfaces of its nanostructures (El-Sherbeeny et al., 2021). The prolonged evaluation period significantly decreased the number of unoccupied sites. The prolonged retention of Sr^2+^ and B^3+^, which exhausts the previously mentioned sites and reduces the overall number of accessible sites, is the most significant factor influencing this reaction. Consequently, there was a notable decrease in the uptake rates of Sr^2+^ and B^3+^ ions after the prescribed intervals. Moreover, the administration of Ca-MCM-41 resulted in negligible improvements or consistent features during the retention of Sr^2+^ and B^3+^, indicating an equilibrium state. The Ca-MCM-41 adsorbent attains its stable state upon complete occupation of all efficient functional sites, preventing further adsorption of strontium and boron ions onto its interface (Abdel Salam et al., 2022).

#### 3.2.3 Kinetic studies

##### 3.2.3.1 Intra-particle diffusion behavior

The examination of intra-particle diffusion characteristics for the adsorption behaviors employing Ca-MCM-41 may elucidate the mechanistic processes and binding affinities of Sr^2+^ and B^3+^. The curves in Figure 5B are divided into three distinct sections, each of which has a distinct slope. The analyzed curves indicate deviations from the initial position, implying the simultaneous presence of several adsorption stages in conjunction with the diffusion characteristics of Sr^2+^ and B^3+^ (El Qada, 2020). The processes generally encompass three main phases: (1) interaction effects of the free surface sites within the Ca-MCM-41 framework (external retention); (2) layered retention types (internal retention) in conjunction with the diffusion characteristics of the dissolving ions; and (3) the impacts of equilibrium or saturation states (Lin et al., 2021). The first findings of the examination suggest that external uptake behaviors primarily promote the binding of Sr^2+^ and B^3+^ within the interfaces of Ca-MCM-41 particulates. This approach performs the most beneficial and predominant regulating effect throughout all stages of the remediation operations (Figure 5B). The effectiveness of the binding activities during this step is determined by the overall quantity of receptors present on the interfaces of the Ca-MCM-41 nanoparticles. The successful operation of the layered or internal adsorption processes as an entirely new removal mechanism was swiftly established by prolonging the duration until all exterior sites were completely saturated (Figure 5B) (Lin et al., 2021; Neolaka et al., 2018). This phase additionally comprises the effects of boron and strontium diffusion mechanisms. After establishing equilibrium, the final stages of Sr^2+^ and B^3+^ binding by Ca-MCM-41 significantly influence the entire mechanism. This stage indicates the successful binding of Sr^2+^ and B^3+^, occupying all available sites (Salam et al., 2020b; Sayed et al., 2022). During this stage, molecular and interionic attraction processes promote the removal of Sr^2+^ and B^3+^ instead of the earlier mentioned pathways (Salam et al., 2020a).

##### 3.2.3.2 Kinetic modeling

Modeling the kinetics of retention behaviors is crucial for examining the physical mechanisms, such as mass transfer events and chemical mechanisms that influence uptake efficiency, while also offering insights into time impacts (Neolaka et al., 2023). The kinetics of Sr^2+^ and B^3+^ reduction was analyzed employing conventional pseudo-first-order (P.F.) and pseudo-second-order (P.S.) modelling approaches. The P.F. model was used to elucidate the correlation between the rate by which Sr^2+^ and B^3+^ completely occupy all binding sites and their entire quantities. The P.S. ideas may elucidate the relationship between the characteristics of analyzed adsorbents during specific temporal periods. The correlation between the binding properties of pollutants and the two analyzed kinetic hypotheses was assessed by nonlinear fitting approaches corresponding to their relevant equations. The analysis of correlation coefficients (*R* ^*2*^) and Chi-squared (*X* ^*2*^) values (Table 1; Figures 5C, D) demonstrated the best degrees of correlation. The *R* ^*2*^ values beside the *X* ^*2*^ data demonstrate that the principal hypotheses of the P.F. model more successfully describe the adsorption characteristics of Sr^2+^ and B^3+^ using Ca-MCM-41 than the P.S. notions. The computational examination of the P.F. model revealed the expected quantities of Sr^2+^ and B^3+^ that Ca-MCM-41 may theoretically adsorb that are 58.45 mg/g and 26.7 mg/g, respectively. These values align with the actually determined amounts in comparison with the results of the P.S. model. The verified agreement confirms previous findings, emphasizing the more effective validity of the P.F. hypothesis in describing strontium and boron uptake tendencies when implementing Ca-MCM-41 (Table 1). P.F. theoretical characteristics suggest that physical aspects, particularly van der Waals forces and electrostatic attractions, strongly impact the binding of Sr^2+^ and B^3+^ onto the exteriors of Ca-MCM-41 nanoparticles (Sherlala et al., 2019; Huang et al., 2018). Although the P.F. model demonstrates a greater degree of alignment, the examined fitting parameters also exhibit considerable matches with the P.S. model. Recent studies suggest that several chemical procedures, including hydrogen bonds and the formation of complexes, may either enhance or have little effect on the elimination of Sr^2+^ and B^3+^ by Ca-MCM-41 (Salam et al., 2020b; Sherlala et al., 2019). Physical approaches may generate successive retention layers over the already formed layers of chemically bound Sr^2+^ and B^3+^ (Jasper et al., 2020).

**TABLE 1:** The mathematical parameters of the evaluated kinetic models.

Kinetic models			
Models	Parameters	Sr^2+^	B^3+^
Pseudo-First-order	K_1_ (min^-1^)	0.0074	0.0056
	Qe _(Cal)_ (mg/g)	58.5	26.7
	R^2^	0.92	0.90
	X^2^	2.07	1.34
Pseudo-Second-order	k_2_ (g mg^-1^ min^-1^)	8.29 × 10^−5^	1.16 × 10^−4^
	Qe _(Cal)_ (mg/g)	75.22	36.6
	R^2^	0.87	0.88
	X^2^	2.82	1.64

#### 3.2.4 Starting concentration

**FIGURE 6:**
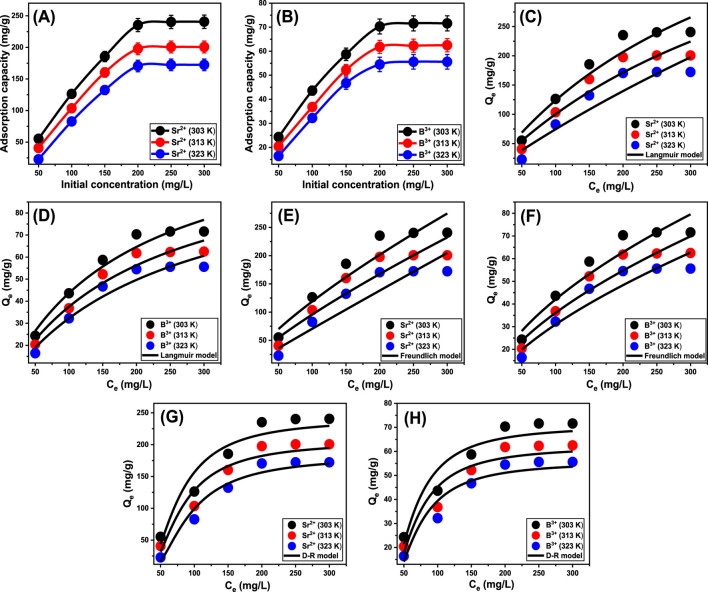
The experimental influence of starting concentrations of Sr^2+^ and B^3+^ on their uptake by Ca-MCM-41 **(A)** (Sr^2+^) and **(B)** (B^3+^), fitting of the uptake behaviors with Langmuir model **(C)** (Sr^2+^) and **(D)** (B^3+^), fitting of the uptake behaviors with Freundlich model **(E)** (Sr^2+^) and **(F)** (B^3+^), and fitting of the uptake behaviors with D-R model **(G)** (Sr^2+^) and **(H)** (B^3+^).

This study investigated the impact of initial Sr^2+^ and B^3+^ concentrations on their maximum elimination capacities utilizing Ca-MCM-41, together with the corresponding equilibrium state, across the evaluated range of 50–300 mg/L. All of the additional essential variables affecting the elimination of Sr^2+^ and B^3+^ were maintained at fixed values: a total volume of 50 mL, an exposure time of 24 h, a Ca-MCM-41 dose of 0.2 g/L, a pH of 9, and temperatures ranging from 303 K to 323 K. A causative relationship can be detected between elevated Sr^2+^ and B^3+^ concentrations and a measurable rise in their sequestered amounts using Ca-MCM-41 (Figures 6A, B). Higher initial concentrations provide a greater concentration gradient, enhancing the mass transfer rate of metal ions from the solution to the adsorbent surface (Jiang et al., 2024). This improved their capacity to interact with a greater number of free and already existing reactive sites across the exterior of Ca-MCM-41. Therefore, the effectiveness of Sr^2+^ and B^3+^ adsorption using Ca-MCM-41 significantly improved in relation to the systematic increment in the metal concentrations. The correlation is detectable only for particular levels of Sr^2+^ and B^3+^. Surpassing these values, rising the starting levels of Sr^2+^ and B^3+^ does not seem to improve their binding effectiveness to the interface of Ca-MCM-41. Determining the equilibrium point is vital for ascertaining the greatest adsorption performance of Sr^2+^ and B^3+^ using Ca-MCM-41. The best achievable adsorption capacities of Sr^2+^ employing Ca-MCM-41 were determined to be 240.6 mg/g at 303 K, 200.3 mg/g at 313 K, and 172.2 mg/g at 323 K (Figure 6A). The determined values for B^3+^ were 71.6 mg/g at 303 K, 62.5 mg/g at 313 K, and 55.6 mg/g at 323 K (Figure 6B). The observed decrease in Sr^2+^ and B^3+^ uptake efficiency using Ca-MCM-41 at higher temperatures indicates that the processes are exothermic.

#### 3.2.5 Classic isotherm models

Traditional equilibrium assessments of adsorption activities were conducted to evaluate the distribution of soluble pollutants inside aqueous solutions that contain adsorbent particles at doses higher than the equilibrium threshold. Conventional equilibrium modeling approaches possess substantial importance to signify the regulating and operating mechanisms. The regularly utilized equilibrium hypotheses yield significant information about (a) the potential selectivity of water-soluble ions throughout the contact area of the utilized adsorbent, (b) the expected quantity of ions that the adsorbent’s surface may capture and adsorb, and (c) the greatest possible retention capacities. The current investigation evaluates the equilibrium properties of Sr^2+^ and B^3+^ throughout their removal by Ca-MCM-41, based on the Langmuir (Figures 6C, D), Freundlich (Figures 6E, F), and Dubinin-Radushkevich (D-R) (Figures 6G, H) isotherm models. The correlation between the assumed equilibrium constraints of the models mentioned earlier and the experimental adsorption properties of Sr^2+^ and B^3+^ was evaluated employing non-linear regression matching methods. The analysis included analyzing the correlation coefficient (*R* ^*2*^) and the Chi-squared (*χ* ^*2*^) parameters. The assessment of *R* ^*2*^ and *χ* ^*2*^ indicates that the Ca-MCM-41 nanoparticles primarily adsorb Sr^2+^ and B^3+^, according to Langmuir’s concept of equilibrium and isotherm rather than the Freundlich hypothesis (Table 2). The established equilibrium feature reveals that Sr^2+^ and B^3+^ ions display homogeneous and consistent behaviors during their uptake by the effective sites along the interface of Ca-MCM-41. Based on these equilibrium properties, the bonded Sr^2+^ and B^3+^ ions form single layers on the surface of Ca-MCM-41 (Huang et al., 2018; Dawodu et al., 2012). Furthermore, the *RL* metrics <1 indicated that the Ca-MCM-41 nanoparticles possessed favorable adsorption properties for Sr^2+^ and B^3+^ ions (El Qada, 2020). The theoretical analysis revealed that Ca-MCM-41 has the maximum Sr^2+^ adsorption capacities (Q_max_) of 320.7 mg/g at 303 K, 309.8 mg/g at 313 K, and 233.4 mg/g at 323 K. The predicted values for B^3+^ are 124 mg/g at 303 K, 112.6 mg/g at 313 K, and 108.1 mg/g at 323 K.

**TABLE 2:** The estimated mathematical parameters of the studied classic equilibrium models.

Pollutant	Model	Parameter	Values		
			303 K	313 K	323 K
Sr^2+^	Langmuir	Q_max_ (mg/g)	320.7	309.8	233.4
		b(L/mg)	0.0025	0.0021	7.1 × 10^−4^
		R^2^	0.95	0.94	0.92
		X^2^	2.75	3.57	4.33
	Freundlich	1/n	0.76	0.81	0.97
		k_F_ (mg/g)	3.56	2.33	0.81
		R^2^	0.92	0.90	0.88
		X^2^	4.33	4.95	5.88
	D-R model	β (mol^2^/kJ^2^)	0.0175	0.0192	0.0247
		Q_m_ (mg/g)	244.8	205.2	182.1
		R^2^	0.95	0.96	0.97
		X^2^	3.41	2.48	1.72
		E (kJ/mol)	5.34	5.10	4.49
B^3+^	Langmuir	Q_max_ (mg/g)	124.0	112.6	108.1
		b(L/mg)	0.0054	0.005	0.0043
		R^2^	0.97	0.97	0.96
		X^2^	0.30	0.35	0.43
	Freundlich	1/n	0.58	0.60	0.64
		k_F_ (mg/g)	2.89	2.26	1.60
		R^2^	0.94	0.93	0.93
		X^2^	0.71	0.74	0.82
	D-R model	β (mol^2^/kJ^2^)	0.0122	0.0129	0.0141
		Q_m_ (mg/g)	74.7	62.1	55.7
		R^2^	0.93	0.93	0.95
		X^2^	0.74	0.73	0.59
		E (kJ/mol)	6.4	6.22	5.95

The equilibrium properties of the D-R model provide an in-depth description of the energetic variations exhibited by Ca-MCM-41 nanostructure throughout the removal processes of Sr^2+^ and B^3+^, irrespective of the particles' levels of heterogeneity or homogeneity. The analysis of the D-R simulation results provides significant information about the adsorption energy (E) that assists in determining the underlying operating mechanisms, whether physical or chemical. Based on their energetic levels, we frequently divide adsorption processes into three distinct groups: below 8 kJ/mol, between 8 and 16 kJ/mol, and over 16 kJ/mol. These energy levels primarily involve strong physical, weak chemical, complex chemical and physical, and extensive chemical mechanisms, respectively (Sayed et al., 2022). The calculated energy (E) values for the adsorption of Sr^2+^ and B^3+^ employing Ca-MCM-41 were within the prescribed energy constraints for physical processes (<8 kJ/mol).

#### 3.2.6 Advanced isotherm models

**FIGURE 7:**
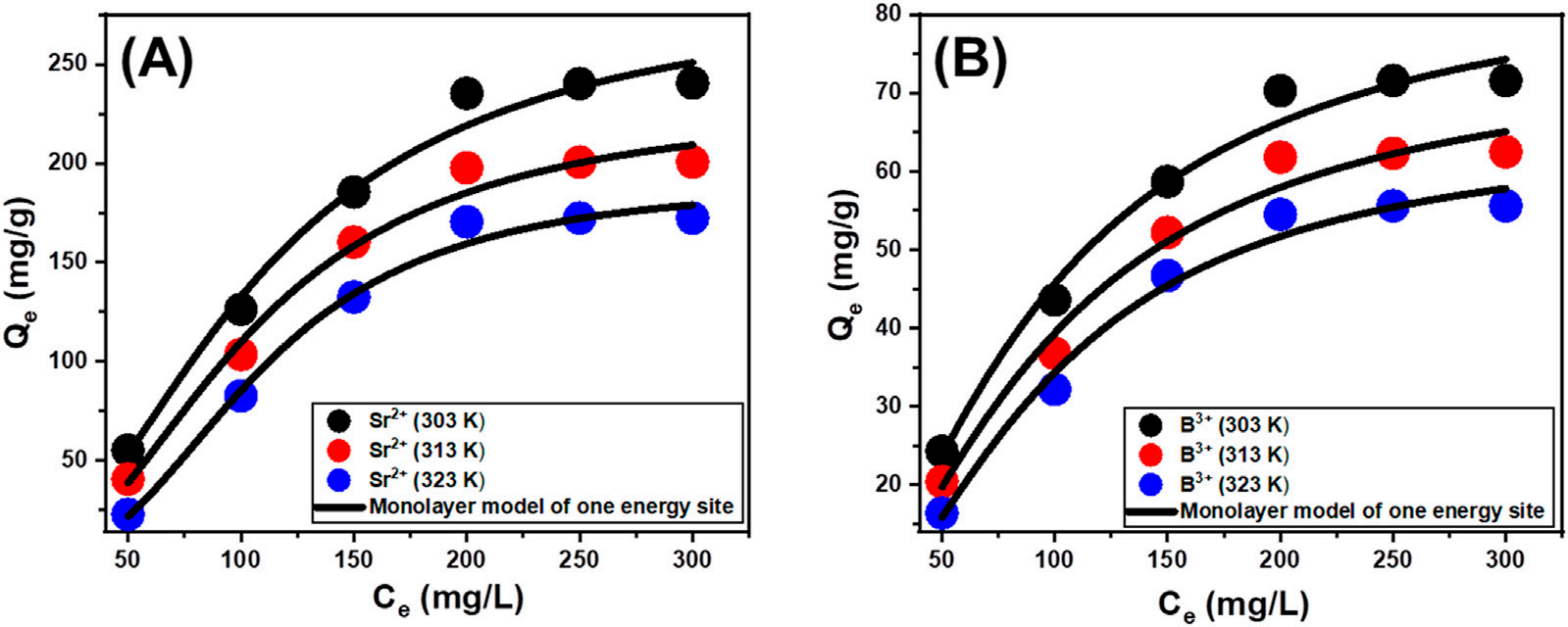
Fitting of the adsorption behaviors of Sr^2+^ **(A)** and B^3+^ **(B)** using Ca-MCM-41 with advanced monolayer equilibrium model of one energy site.

A statistical physics model, which describes the equilibrium aspects of the adsorption tendencies, may provide a detailed examination of the distinctive characteristics of these kinds of reactions. The reactions between water-soluble pollutants and the most predominant reactive chemical groups that serve as receptors over the absorbent’s surfaces are analyzed employing the mathematical simulations of these advanced models. The mathematical equations employed throughout this investigation provide reliable approximate measurements that adequately reflect the main mechanistic processes, encompassing both steric and energetic factors. The main studied steric factors include Nm, which is the total quantity of occupied adsorption sites throughout the exterior of Ca-MCM-41, the total number of metal ions anchored (n) into an individual receptor, and the maximum possible adsorption capacities of Sr^2+^ and B^3+^ using Ca-MCM-41 when they reach their full saturation (Q_sat_). Internal energy (E_int_), entropy (Sa), adsorption energy (E), and free enthalpy (G) are the investigated energetic and thermodynamic elements. Non-linear regression assessment was used to analyze the fitting approaches using the hypotheses of the earlier models. This investigation finished successfully utilizing the Levenberg-Marquardt iterative approach in conjunction with multivariable nonlinear regression methods. The adsorption characteristics of Sr^2+^ and B^3+^ via Ca-MCM-41 were evaluated and discussed based on the determined fitting levels. The monolayer model of a single active site was implemented during the assessment based on the recognized fitting levels (Figures 7A, B; Table 3). Table 3 displays the calculated values for the predicted fitting factors for the used model.

**TABLE 3:** The estimated mathematical parameters of advanced isotherm models.

		n	Nm (mg/g)	Q_sat_ (mg/g)	C_1/2_ (mg/L)	ΔE (kJ/mol)
Sr^2+^	303K	1.92	148.9	285.9	101.8	−5.81
	313K	2.17	106.2	230.5	104.4	−6.10
	323K	2.64	72.5	191.3	108.5	−6.40
B^3+^	303K	1.57	54.8	86.1	92.4	−3.62
	313K	1.63	45.8	74.7	93.8	−3.77
	323K	1.79	36.2	64.8	94.4	−3.91

**TABLE 4:** Comparison between Ca-MCM-41 and other adsorbents.

Adsorbent	Pollutant	Q_max_ (mg/g)	References
T-resin	B^3+^	21.25	Bai et al. (2020)
Al_2_O_3_/calcium alginate	B^3+^	56.3	Demey et al. (2019)
Glycidol@MgFe_2_O_4_	B^3+^	68.9	Oladipo and Gazi (2016)
Mg–Al–CLDHs nanosheet	B^3+^	77.8	Gao et al. (2017)
Fly ash	B^3+^	46.2	Öztürk and Kavak (2005)
Sugarcane bagasse biochar	B^3+^	36	Liao et al. (2024)
Pure MCM-41	B^3+^	58.4	This study
Ca-MCM-41	B^3+^	86.1	This study
Chitosan-Alginate Beads	Sr^2+^	29	Hasan et al. (2019)
Plant-Derived Activated Carbon	Sr^2+^	8.34	Ceban et al. (2023)
Nanoporous Aluminoborate Spheres	Sr^2+^	163.08	Abbasi et al. (2024)
Zeolitic Material from Volcanic Rock	Sr^2+^	154.8	Lee et al. (2021)
PANI@GO	Sr^2+^	149.52	Sun et al. (2013)
Nanoscale activated biochar	Sr^2+^	59.8	Younis et al. (2020)
Pure MCM-41	Sr^2+^	184.7	This study
Ca-MCM-41	Sr^2+^	285.9	This study

##### 3.2.6.1 Steric properties

###### 3.2.6.1.1 Number of adsorbed ions per site (n)

**FIGURE 8:**
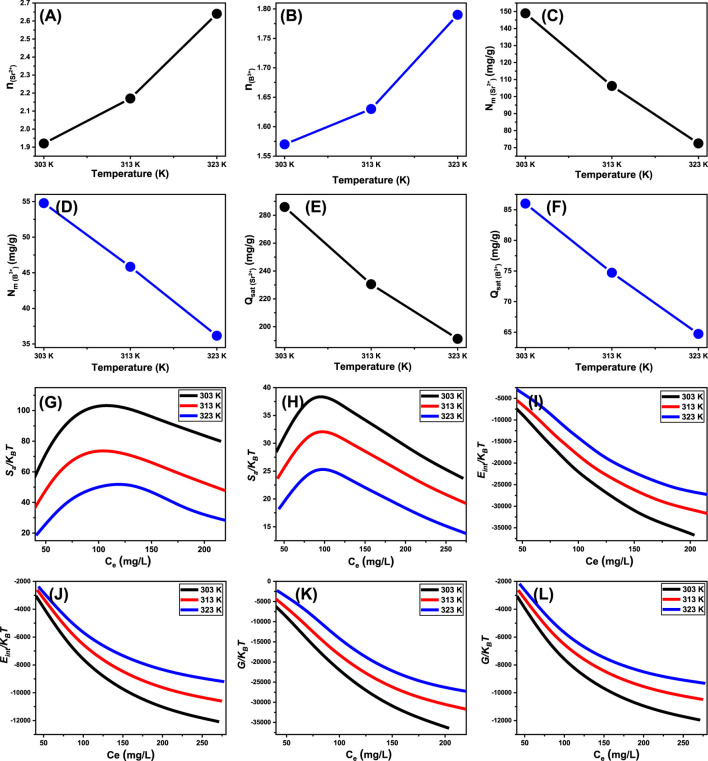
Changes in the steric and thermodynamic functions during the adsorption reactions using Ca-MCM-41 including number of adsorbed ions per site **(A)** (Sr^2+^) and **(B)** (B^3+^), active site density **(C)** (Sr^2+^) and **(D)** (B^3+^), saturation uptake capacity **(E)** (Sr^2+^) and **(F)** (B^3+^), entropy **(G)** (Sr^2+^) and **(H)** (B^3+^), internal energy **(I)** (Sr^2+^) and **(J)** (B^3+^), and enthalpy **(K)** (Sr^2+^) and **(L)** (B^3+^).

The theoretical analysis of the n value provides adequate details about the spatial distribution of anchored Sr^2+^ and B^3+^ onto the exterior interface of Ca-MCM-41. This relevance includes both vertical and horizontal arrangements. Moreover, these insights are essential for understanding the mechanisms that affect the uptake activities, which comprises numerous dockings or interactions. The uptake of just one Sr^2+^ and B^3+^ ion via various retention receptors is significantly affected by the presence of multi-anchorage or multi-docking processes. Such binding pathways have levels less than 1, demonstrating the horizontal configuration of these adsorbed ions. Conversely, reactions with values beyond a magnitude more than one indicate the existence of Sr^2+^ and B^3+^ ions in non-parallel arrangements, coupled by vertical alignments. Multi-ionic interactions usually control elimination processes of such systems, allowing a single site to accommodate several ions (Sayed et al., 2022; Mobarak et al., 2021). The calculated values of n, denoting the total quantity of Sr^2+^ and B^3+^ ions occupying a single uptake site onto the exterior of the Ca-MCM-41 product, ranged from 1.92 to 2.64 for Sr^2+^ (Figure 8A) and from 1.57 to 1.79 for B^3+^ (Figure 8B). The total quantity of Sr^2+^ and B^3+^ ions associated with each site exceeds one. As a result, multi-ionic interaction mechanisms significantly regulated the retention of Sr^2+^ and B^3+^ ions. Each binding receptor across the contact surface of Ca-MCM-41 may accommodate up to three ions of Sr^2+^ and only two ions of B^3+^, organized in vertical configurations with non-parallel characteristics. The n measurements for Ca-MCM-41 demonstrate a substantial rise in relation to the examined temperature conditions. This signifies an enhancement in the aggregation properties of the two ions during their collisions with the exterior of Ca-MCM-41 product. This also demonstrates the impact of thermal activation activities that could transpire prior to the adsorption of Sr^2+^ and B^3+^ ions (Mobarak et al., 2021; Dhaouadi et al., 2021).

###### 3.2.6.1.2 Density of the active sites (Nm)

Assessing the quantity of functional retention sites for Sr^2+^ and B^3+^ (Figures 8C, D) could yield a quantitative estimate for the entire quantity of sites filled with Sr^2+^ and B^3+^ (Nm) across the interaction interfaces of Ca-MCM-41 particles. The Nm values for Sr^2+^ at different temperatures are 148.9 mg/g at 303 K, 106.2 mg/g at 313 K, and 72.5 mg/g at 323 K (Figure 8C). The detected values for B^3+^ are 54.8 mg/g at 303 K, 45.8 mg/g at 313 K, and 36.2 mg/g at 323 K (Figure 8D). These results elucidate the better adsorption efficacy of Ca-MCM-41 for Sr^2+^ and B^3+^ ions. The occupied sites on the surface of Ca-MCM-41 during the uptake of Sr^2+^ and B^3+^ exhibit temperature-dependent reversible variation (Figures 8C, D). The adverse impact of temperature on the activation levels of the described retention sites may explain the observed patterns (Dehkordi et al., 2024; Sellaoui et al., 2020). The analysis highlights the detrimental effects of rising temperatures on the quantity and nature of occupied sites, as illustrated by the reduction in the functional sites or the decreased suitable contact time necessary for these sites to hold and retain Sr^2+^ and B^3+^ ions. Prior research has attributed these behaviors to the predicted desorption of adsorbed ions and their escape from the surface of Ca-MCM-41. The reduction in saturation limitations in warm solutions led to enhancements in desorption behaviors (Ahmed et al., 2024).

###### 3.2.6.1.3 Adsorption capacity at the saturation state of (Q_sat_)

The entirely saturated adsorption capacity of Ca-MCM-41 (Q_sat_) demonstrates the most effective strontium and boron uptake performance with the highest tolerance aspects. Two primary factors affect the estimated quantity of Q_sat_: the predefined density of occupied sites (Nm) and the total number of ions filling each site (n) individually. Ca-MCM-41 exhibits saturation adsorption capabilities for Sr^2+^ of 285.9 mg/g at 303 K, 230.5 mg/g at 313 K, and 191.3 mg/g at 323 K (Figure 8E). The determined values for B^3+^ were 86.1 mg/g at 303 K, 74.7 mg/g at 313 K, and 64.8 mg/g at 323 K (Figure 8F). The negative effects of temperature demonstrate the exothermic characteristics of the Sr^2+^ and B^3+^ elimination processes using Ca-MCM-41. The findings indicate that elevated adsorption temperature trigger the thermal collisions processes, hence reducing the binding efficacy of Sr^2+^ and B^3+^ (Sayed et al., 2022). Moreover, the temperature-dependent observable properties of Q_sat_ resemble the behavior associated with Nm, instead of n. The results reveal that the total number of interacting sites, rather than the particular binding capacity of individual receptors, is the key factor influencing the efficacy of Sr^2+^ and B^3+^ retention.

##### 3.2.6.2 Energetic properties

###### 3.2.6.2.1 Adsorption energy

The analysis of variations in energy (ΔE) during the binding of Sr^2+^ and B^3+^ ions provides valuable insights into the basic mechanisms, regardless of their association with chemical or physical processes. Physical processes show energies below 40 kJ/mol, while chemical pathways have energetic levels over 80 kJ/mol. These binding energies serve as key parameters for classifying different kinds of physical mechanisms. The physical processes outlined include hydrogen bonds (<30 kJ/mol), dipole bonding impacts (2–29 kJ/mol), van der Waals forces (4–10 kJ/mol), electrostatic attraction (2–50 kJ/mol), and hydrophobic bonding (=5 kJ/mol). The adsorption energy estimates (ΔE) for Sr^2+^ and B^3+^ ions were calculated using Equation 5. This equation includes the solubility of the Sr^2+^ and B^3+^ (S), the gas constant (R = 0.008314 kJ/mol·K), and the levels of Sr^2+^ and B^3+^ ions that exist during the half-saturation conditions of Ca-MCM-41, as well as a specific temperature (T) (Dhaouadi et al., 2020).

$$∆E=RT\;\ln\left(\frac{S}{C}\right)$$ (5)

The Ca-MCM-41 framework demonstrates Sr^2+^ adsorption energies ranging from −5.8 to −6.4 kJ/mol and from −3.6 to −3.9 kJ/mol for B^3+^ ions (Table 3). Such limits are acknowledged constraints for physisorption mechanisms. The quantitative findings demonstrate that hydrogen-bonding, electrostatic attraction, dipole interactions, and van der Waals forces are the main physical processes facilitating the removal of both Sr^2+^ and B^3+^ ions using Ca-MCM-41. The negative ∆E measurements indicate that the interaction activities of Sr^2+^ and B^3+^ ions were exothermic.

###### 3.2.6.2.2 Thermodynamic functions

####### 3.2.6.2.2.1. Entropy

The entropy (Sa) properties corresponding to the adsorption reactions of Sr^2+^ and B^3+^ ions employing Ca-MCM-41 clearly illustrate the ordered and disordered properties of the adsorbent’s exterior interfaces under different levels of Sr^2+^ and B^3+^ ions and various temperature conditions. The distinctive features of Sa can be evaluated using the results derived from Equation 6, utilizing the earlier determined values for Nm and n, in addition to the expected content of Sr^2+^ and B^3+^ ions during the half-saturation states of Ca-MCM-41 (C1/2) (Sellaoui et al., 2020; Dhaouadi et al., 2020).

$$\frac{S_{a}}{K_{B}}=Nm\left\{\ln\left(1+{\left(\frac{C}{C_{\frac{1}{2}}}\right)}^{n}\right)-n{\left(\frac{C}{C_{\frac{1}{2}}}\right)}^{n}\;\frac{\ln\left(\frac{C}{C_{\frac{1}{2}}}\right)}{1+{\left(\frac{C}{C_{\frac{1}{2}}}\right)}^{n\;}}\;\right\}$$ (6)

The analysis of the resultant graphs reveals a notable decrease in entropy degrees (Sa) following the interaction of Sr^2+^ and B^3+^ ions with Ca-MCM-41, especially at higher levels of their ions (Figures 8G, H). The studies reveal a significant reduction in the disorder features of the Ca-MCM-41 interface with elevated concentrations of the studied Sr^2+^ and B^3+^ ions. The entropy features furthermore indicate the enhancement in the efficacy of Sr^2+^ and B^3+^ docking at the unfilled binding sites situated on the Ca-MCM-41 exterior, even in the existence of low contents of these ions (Sellaoui et al., 2020; Dhaouadi et al., 2020). The peak entropy degrees following Sr^2+^ adsorption were established at equilibrium concentrations of 105.8 mg/L (303 K), 120.9 mg/L (313 K), and 131.8 mg/L (323 K) (Figure 8G). The equilibrium levels of B^3+^ ions, corresponding to the maximum entropy, were 82.5 mg/L at 303 K, 85.3 mg/L at 313 K, and 87.2 mg/L at 323 K (Figure 8H). These equilibrium concentrations closely correspond to those established with Ca-MCM-41 during the half-saturation phases. Consequently, the binding of additional ions onto the leftover uptake sites is impeded. The significant decreases in the observed entropy levels demonstrate a substantial reduction in the overall number of available sites. This is followed by a significant reduction in the mobility and diffusion of Sr^2+^ and B^3+^ ions (Sellaoui et al., 2016).

####### 3.2.6.2.2.2. Internal energy and free enthalpy

This study evaluated the internal energy (E_int_) correlated with the removal of strontium and boron ions by Ca-MCM-41, in conjunction with the free enthalpy (G), while encompassing variations in Sr^2+^ and B^3+^ contents and the effects of system temperature. The assessment was performed using Equations 7, 8 based on the determined values for Nm, n, and C1/2, together with the translational partition (Zv) (Dhaouadi et al., 2021).

$$\frac{E_{\mathrm{int}}}{K_{B}T\;}=n\;N_{m}\left[\left(\;\frac{{\left(\frac{C}{C_{1/2}}\right)}^{n}\;\ln\left(\frac{C}{Z_{v}}\right)}{1+{\left(\frac{C}{C_{1/2}}\right)}^{n}}\right)-\left(\;\frac{n\ln\left(\frac{C}{C_{1/2}}\right)\;{\left(\frac{C}{C_{1/2}}\right)}^{n}}{1+{\left(\frac{C}{C_{1/2}}\right)}^{n}}\right)\right]$$ (7)

$$\frac{G}{K_{B}T\;}=n\;N_{m}\frac{\ln\left(\frac{C}{Z_{v}}\right)}{1+{\left(\frac{C_{1/2}}{C}\right)}^{n}}$$ (8)

The evaluated changes in E_int_ with respect to the removal processes of Sr^2+^ and B^3+^ ions using Ca-MCM-41 exhibit negative values. The results demonstrated a significant reduction in E_int_ as the temperature increased from 303 K to 323 K (Figures 8I, J). This investigation confirms the spontaneity and exothermic aspects of the adsorption reactions using Ca-MCM-41. The enthalpy assessments exhibit similar characteristics to the behaviors observed during the internal energy assessment. The G results demonstrate a reversible relationship with the prescribed adsorption temperature (Figures 8K, L). This signifies a decrease in feasible characteristics and corroborates the exothermic and spontaneous features of the adsorption reactions of Sr^2+^ and B^3+^ ions utilizing Ca-MCM-41.

#### 3.2.7 Recyclability

The recycling aspect is a vital factor for marketing as well as the actual application of produced Ca-MCM-41 particles. The regeneration procedures included washing the Ca-MCM-41 particles across multiple rounds using distilled water, with each round extending 15 min. The washed particles were dried in an electric programmed drier at 60°C over 12 h for reuse in the following phase of the Sr^2+^ and B^3+^ adsorption processes. The laboratory factors influencing the removal of Sr^2+^ and B^3+^ were held constant: a total volume of 50 mL, treatment duration of 24 h, a dosage of 0.2 g/L, a pH of 9, a specific concentration of 300 mg/L, and a temperature of 303 K. The developed Ca-MCM-41 structure exhibits significant reusability and recycling capacity across the five analyzed reusing rounds. The Sr^2+^ removal efficiency surpasses 235 mg/g for two cycles, 218 mg/g for four cycles, and 200 mg/g for five cycles (Supplementary Figure S3). During B^3+^ retention, Ca-MCM-41 demonstrated recyclability with removal capacities exceeding 70 mg/g for two rounds, more than 60 mg/g in four rounds, and beyond 53 mg/g in five rounds (Supplementary Figure S3).

#### 3.2.8 Comparison study

The adsorption performances of the Ca-MCM-41 were compared with pure phase of MCM-41 and other investigated materials in literature. The comparison study shows that Ca-MCM-41 exhibits significantly higher adsorption capacities for both B^3+^ (boron ions) and Sr^2+^ (strontium ions) compared to pure MCM-41. The data shows that Ca-MCM-41 has a Q_max_ of 86.1 mg/g for B^3+^ and 285.9 mg/g for Sr^2+^, compared to 58.4 mg/g and 184.7 mg/g for pure MCM-41, respectively. The significant improvement in adsorption capacity is likely due to:

• Increased ion-exchange capability: The incorporation of calcium enhances the material’s affinity toward cationic pollutants (e.g., Sr^2+^) through electrostatic interactions.
• Structural modifications: Ca^2+^ may stabilize surface functional groups, leading to better adsorption efficiency.
• Higher surface charge density: Ca doping likely modifies the pore environment, increasing active sites for adsorption.

Moreover, Ca-MCM-41 demonstrates better adsorption performance compared to pure several investigated adsorbents in literature, making it a promising candidate for real-world applications in water purification, environmental remediation, and industry. Its commercialization potential is strong due to high adsorption capacity, cost-effectiveness, and reusability. Future research should focus on large-scale production, regeneration studies, and hybrid materials for enhanced performance.

#### 3.2.9 Realistic study

The Ca-MCM-41 composite was utilized for the realistic sequestration of sulfate ions from a representative seawater sample collected along the Gulf of Suez coast. Additionally, its efficiency in the direct uptake of strontium (Sr^2+^) and boron (B^3+^) ions was assessed under varying dosage conditions, ranging from 0.4 g/L to 2 g/L. The experiments were conducted under controlled conditions, maintaining an exposure time of 24 h, a room temperature of 33 °C, and a sample volume of 1,000 mL. The initial concentrations of Sr^2+^ and B^3+^ in the seawater were determined to be 24.2 mg/L and 12.85 mg/L, respectively and the pH value was determined during the test to be 7.6. The findings underscore the high sequestration efficiency of the Ca-MCM-41 framework, synthesized from coral reef-derived precursors, in effectively removing Sr^2+^ and B^3+^ from real seawater. Increasing the Ca-MCM-41 dosage significantly enhanced the removal efficiency, with the application of 2 g/L resulting in an 80.2% reduction of Sr^2+^ (from 24.2 mg/L) (Table 5). This corresponds to an adsorption capacity of 235 mg of Sr^2+^ per Gram of Ca-MCM-41, with potential for re-extraction via chemical or physical techniques or the formation of strontium-bearing silicate nanostructures for alternative applications. Similarly, the Ca-MCM-41 composite effectively eliminated 64.2% of B^3+^ (from 12.85 mg/L). Each Gram of Ca-MCM-41 could accommodate up to 109 mg of boron ions, presenting a commercially valuable potential for re-extracting physically bonded boron. These results are highly promising, particularly considering the investigated sample volume and the presence of coexisting chemical ions, further highlighting the remarkable selectivity of Ca-MCM-41 for Sr^2+^ and B^3+^ removal.

**TABLE 5:** Realistic adsorption of strontium and boron ions from seawater using Ca-MCM-41.

Dose (g/L)	Remaining (mg/L)	Removal (%)	Capacity (mg/g)
Sr^2+^			
Control	24.2 mg/L	-------	-------
0.4 g/L	19.7 mg/L	18.6%	225 mg/g
0.8 g/L	14.8 mg/L	38.8%	235 mg/g
1.2 g/L	10.3 mg/L	57.4%	231.7 mg/g
1.6 g/L	7.4 mg/L	69.4%	210 mg/g
2 g/L	4.8 mg/L	80.2%	194 mg/g
B^3+^			
Control	12.85 mg/L	-------	-------
0.4 g/L	11.3 mg/L	12.8%	77.5 mg/g
0.8 g/L	9.4 mg/L	26.8%	86.3 mg/g
1.2 g/L	6.3 mg/L	50.9%	109.2 mg/g
1.6 g/L	5.1 mg/L	60.3%	96.8 mg/g
2 g/L	4.6 mg/L	64.2%	82.5 mg/g

## 4 Conclusion

Highly porous Ca-MCM-41 structure was successfully prepared based on ancient coral reefs with a surface area of 159.6 m^2^/g as a potential adsorbent for Sr^2+^ and B^3+^ ions. The products achieved significant saturation uptake performances of 285.9 mg/g (Sr^2+^) and 86.1 mg/g (B^3+^). The uptake processes were explained according to the principles of Langmuir traditional isotherm and advanced monolayer isotherm model of one energy site. The structure exhibits higher quantities of active uptake receptors for Sr^2+^ (Nm = 148.9 mg/g) than for B^3+^ (Nm = 54.8 mg/g). Also, each existed receptor can adsorb up to 3 Sr^2+^ ions and 2 B^3+^ ions in vertical ordering and by multi-ionic interaction mechanisms. These mechanisms are related to physical processes with uptake energies <7 kJ/mol that occurred spontaneously with exothermic properties. The structure can be effectively used in the sequestration of the two ions (24.2 mg/g (Sr^2+^) and 12.85 (B^3+^)) from seawater along the Gulf of Suez with an adsorption efficiency of 80% (Sr^2+^) and 64% (B^3+^). This recommends the application of the Ca-MCM-41 structure to be used in the decontamination of Sr^2+^ and B^3+^ from industrial wastewater or the recovery of them from seawater.

## Data Availability

The original contributions presented in the study are included in the article/Supplementary Material, further inquiries can be directed to the corresponding author.
