# Supplementary material for: Effective and realistic sequestration of Sr2+ and B3+ ions from the aqueous environments using coral reefs based Ca-MCM-41: Gulf of Suez as case study
Source: Front Chem. 2025 Apr 16;13:1550726. doi: 10.3389/fchem.2025.1550726 (PMC12041029; doi:10.3389/fchem.2025.1550726)
Supplement: Supplementary file 1 [file DataSheet1.docx]

Table.S1. Nonlinear equations of kinetic, classic isotherm, and advanced isotherm models

| Kinetic models | | |
| --- | --- | --- |
| Model | Equation | Parameters |
| Pseudo-first-order | $Q_{t}=Q_{e} (1-e^{{-k}_{1}.t})$ | Q_t_ (mg/g) is the adsorbed ions at time (t), and K_1_ is the rate constant of the first-order adsorption (1/min) |
| Pseudo-second-order | $Q_{t}=\frac{Q_{e}^{2}k_{2}t}{1+Q_{e}k_{2}t}$ | Qe is the quantity of adsorbed ions after equilibration (mg/g), and K_2_ is the model rate constant (g/mg min). |
| Classic Isotherm models | | |
| Model | Equation | Parameters |
| Langmuir | $Q_{e}=\frac{Q_{max} bC_{e}}{(1+bC_{e})}$ | *C_e_* is the rest ions concentrations (mg/L), *Q_max_* is the theoritical maximum adsorption capacity (mg/g), and *b* is the Langmuir constant (L/mg) |
| Freundlich | $Q_{e}=K_{f}C_{e}^{1/n}$ | K_F_ (mg/g) is the constant of Freundlich model related to the adsorption capacity and n is the constant of Freundlich model related to the adsorption intensities |
| Dubinin–Radushkevich | $Q_{e}=Q_{m}e^{-\betaɛ^{2}}$ | β (mol^2^/KJ^2^) is the D-R constant, ɛ (KJ^2^/mol^2^) is the polanyil potential, and Q_m_ is the adsorption capacity (mg/g) |
| Advanced isotherm models | | |
| Model | Equation | Parameters |
| Monolayer model with one energy site (Model 1) | $Q=nN_{o} =\frac{nN_{M}}{1+{(\frac{C1/2}{C})}^{n}}=\frac{Q_{o}}{1+{(\frac{C1/2}{C})}^{n}}$ | Q is the adsorbed quantities in mg/g  n is the number of adsorbed ion per site  Nm is the density of the effective receptor sites (mg/g)  Q_o_ is the adsorption capacity at the saturation state in mg/g  C1/2 is the concentration of the ions at half saturation stage in mg/L  C1 and C2 are the concentrations of the ions at the half saturation stage for the first active sites and the second active sites, respectively  n1 and n2 are the adsorbed ions per site for the first active sites and the second active sites, respectively |
| Monolayer model with two energy sites (Model 2) | $Q=\frac{n_{1}N_{1M}}{1+{(\frac{C_{1}}{C})}^{n_{1}}}+\frac{n_{2}N_{2M}}{1+{(\frac{C_{2}}{C})}^{n_{2}}}$ |  |
| Double layer model with one energy site (Model 3) | $Q=Q_{o}\frac{({\frac{C}{C1/2})}^{n}+2({\frac{C}{C1/2})}^{2n}}{1+({\frac{C}{C1/2})}^{n}+({\frac{C}{C1/2})}^{2n}}$ |  |
| Double layer model with two energy sites (Model 3) | $Q=Q_{o}\frac{({\frac{C}{C1})}^{n}+2({\frac{C}{C2})}^{2n}}{1+({\frac{C}{C1})}^{n}+({\frac{C}{C2})}^{2n}}$ |  |


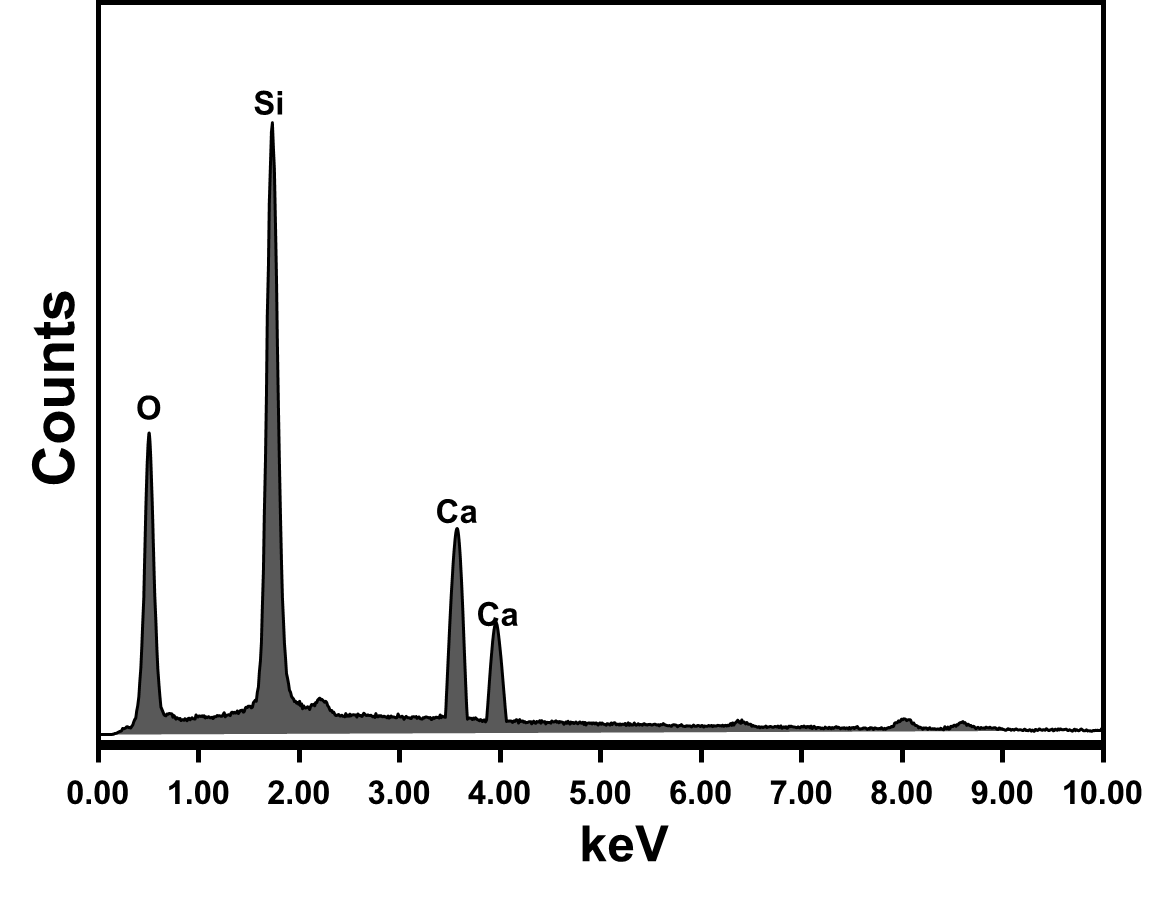


**Fig.S1.** The EDX spectrum of Ca-MCM-41


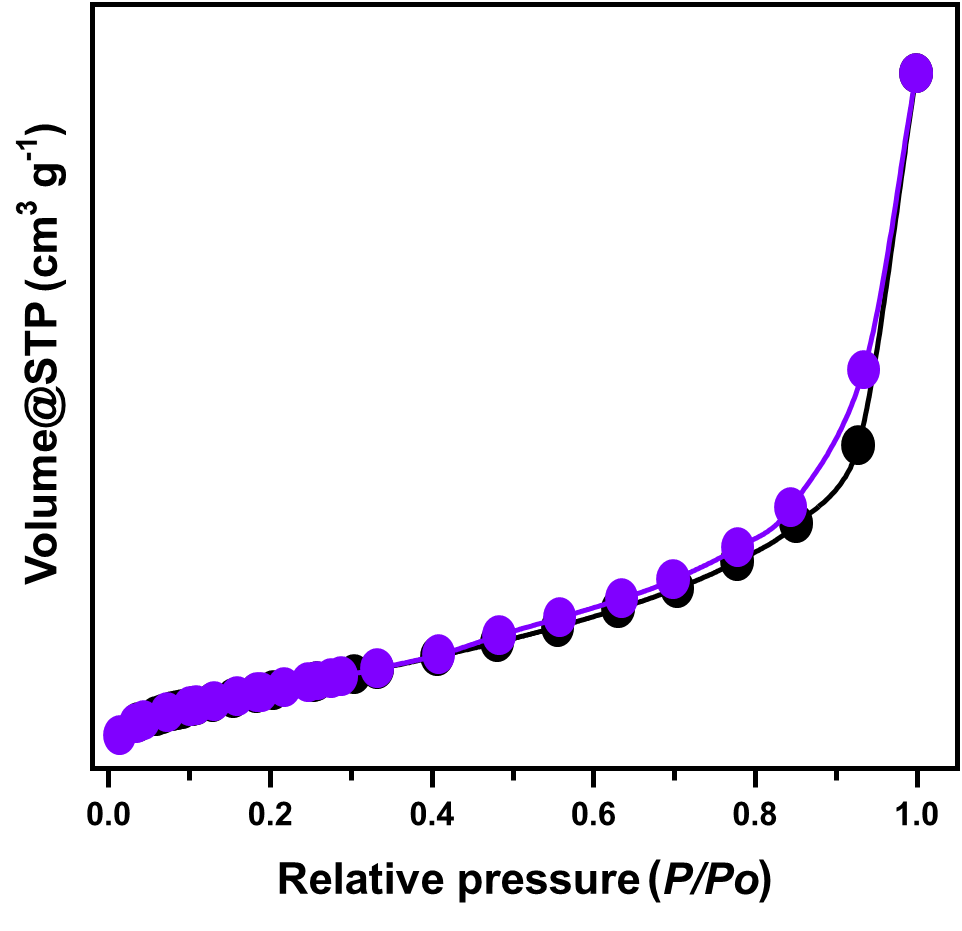


**Fig.S2.** The obtained nitrogen adsorption/desorption isotherm curve of Ca-MCM-41

**Fig.S3.** The recyclability properties of Ca-MCM during the adsorption of strontium and boron ions
